# Supplementary material for: Proton and Carbon Ion Irradiation Changes the Process of Endochondral Ossification in an Ex Vivo Femur Organotypic Culture Model
Source: Cells. 2023 Sep 18;12(18):2301. doi: 10.3390/cells12182301 (PMC10527791; doi:10.3390/cells12182301)
Supplement: Supplementary file 1 [file cells-12-02301-s001.zip › cells-2490794-supplementary.pdf]

Supplementary data

Table S1. List of qPCR primers with respective forward (fw) and reverse (rev) primer sequence.

Target	**Primer**	**Sequence 5'-3'**
glyceraldehyde-3-phosphate dehydrogenase (GAPDH)	fw	CCT-GCA-CCA-CCA-ACT-GCT-TAG-C
	rev	GCC-AGT-GAG-CTT-CCC-GTT-CAG-C
hypoxanthine phosphoribosyltransferase 1(HPRT1)	fw	GCG-AAA-GTG-GAA-AAG-CCA-AGT
	rev	GCC-ACA-TCA-ACA-GGA-CTC-TTG-TAG
collagen type II alpha 1 chain (Col2α1)	fw	GGCCAGGATGCCCGAAAATTA
	rev	GTCACCTCTGGGTCCTTGTTC
collagen type X alpha 1 chain (ColX)	fw	CCTCTGAGCACCAGAATCCA
	rev	CGTCAAGGACACTAGCAGCA
Bone morphogenetic protein 2 (BMP-2)	fw	AAGCCAAACACAAACAGCGG
	rev	GCCACGATCCAGTCATTCCA
runt-related transcription factor 2 (Runx2)	fw	GCGCATTCCTCATCCCAGTA
	rev	GGTGGGGAGGATTGTGTCTG
Osteoprotegerin (OPG)	fw	CTCACTTGGCCTCCTGCTAA
	rev	TCGCACAGGGTGACATCTAT
Osteopontin (SPP1)	fw	CCAGCCAAGGACCAACTACA
	rev	AGTGTTTGCTGTAATGCGCC
Alkaline Phosphatase (ALPL)	fw	CGGTTGGTAGCCTCCTTCTG
	rev	GACGTTCCGATCCTGAGTGG

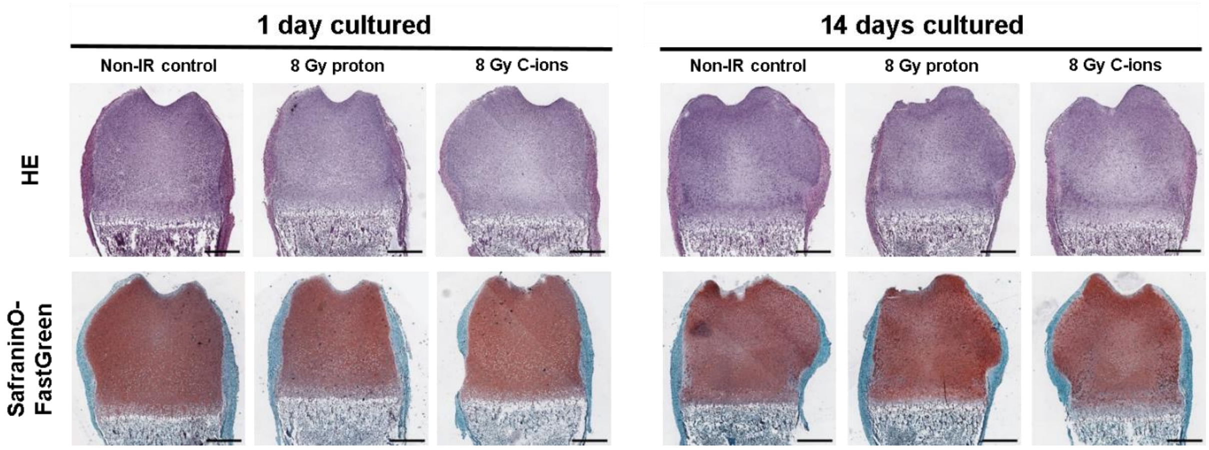

Figure S1. Overview of distal end of the femur histologically stained. Representative images of Haematoxylin&Eosin (H&E) and SafraninO-Fast Green staining of *ex vivo* bone cultures exposed to either 8 Gy proton or C-ions radiation with their respective controls followed by subsequent 1- and 14- days *in vitro* (DIV) cultivation. Scale bar – 500 μm.

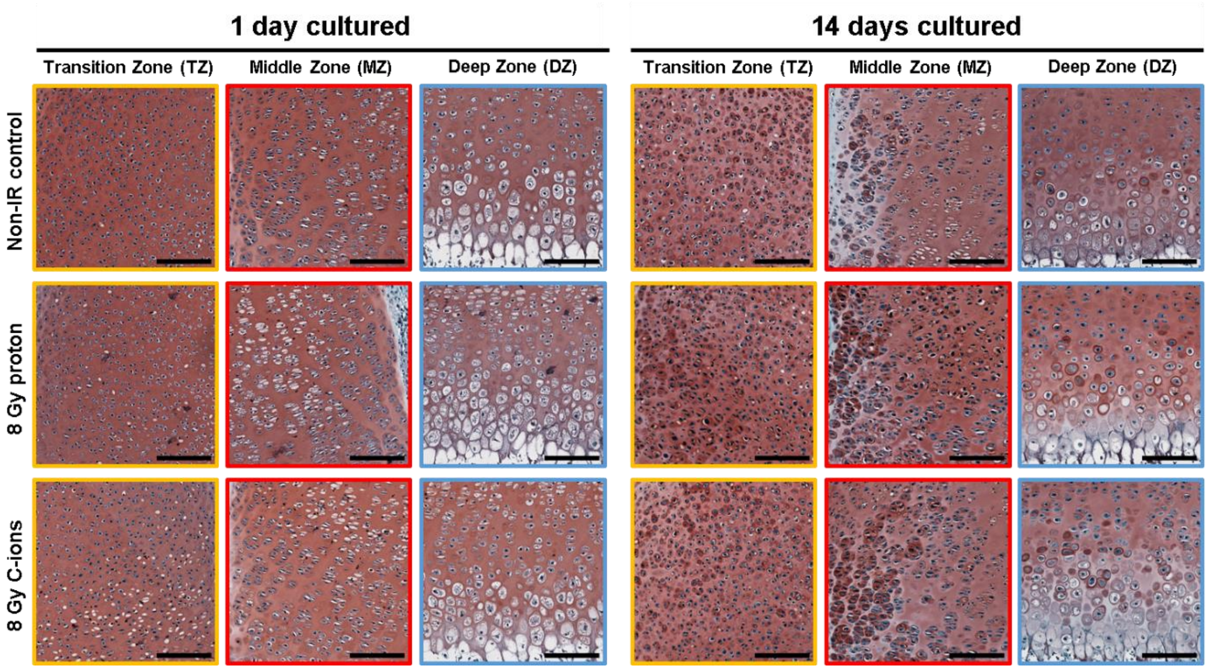

Figure S2. Increased proteoglycan production due to radiation. Representative images of Safranin O-Fast Green staining of *ex vivo* bone cultures exposed to either 8 Gy proton or C-ions radiation with their respective controls followed by subsequent 1- and 14- days *in vitro* (DIV) cultivation. Coloured squares represent transition zone (TZ, yellow), middle zone (MZ, red) and deep zone (DZ, blue). Scale bar - TZ/MZ/DZ = 150 μm.

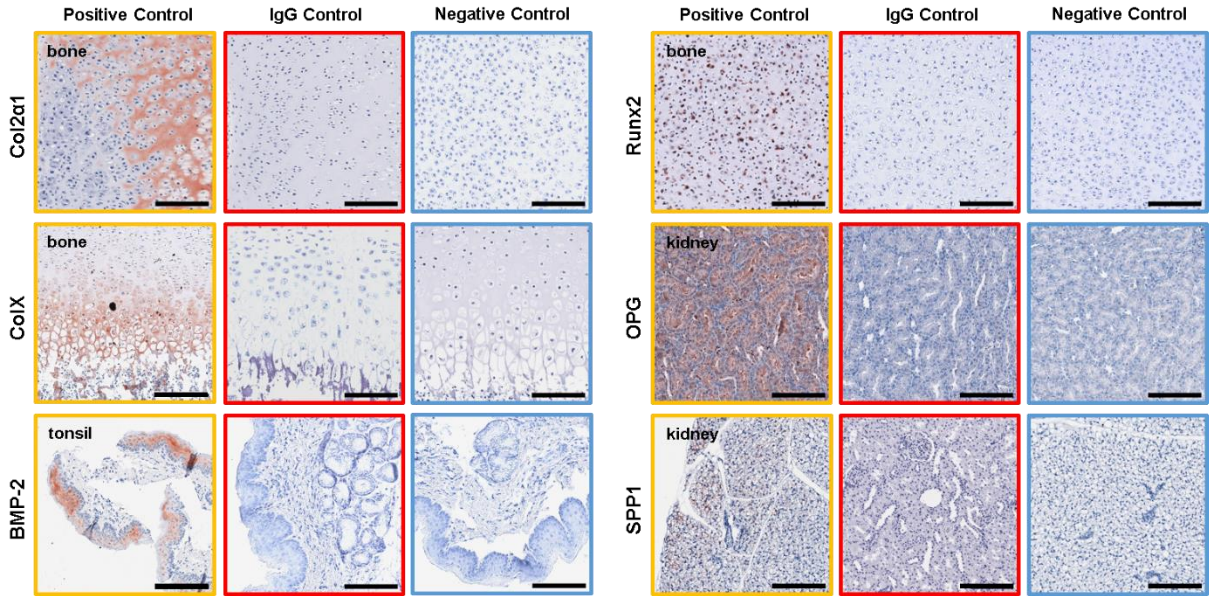

Figure S3. Immunohistochemistry controls. Positive control, IgG control – IgG antibody with respective concentration of primary antibody and negative control – only antibody dilution of appropriate tissue for Col2αa, ColX, BMP-2, Runx2, OPG, and SPP1 are presented. Scale bar = 150 μm.

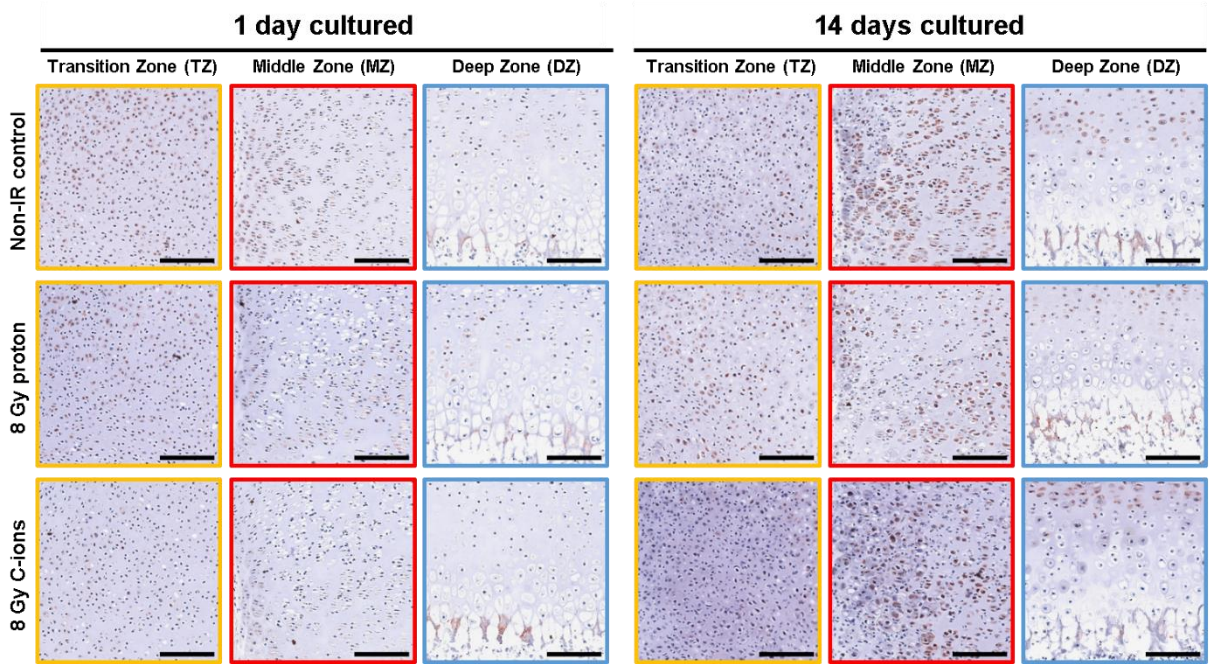

Figure S4. Immunohistochemistry of Bone morphogenetic factor 2 (BMP-2). Representative images of BMP-2 IHC of *ex vivo* bone cultures exposed to either 8 Gy proton or C-ions radiation with their respective controls followed by subsequent 1- and 14- days *in vitro* (DIV) cultivation. Coloured squares represent transition zone (TZ, yellow), middle zone (MZ, red) and deep zone (DZ, blue). Scale bar - TZ/MZ/DZ = 150 μm.

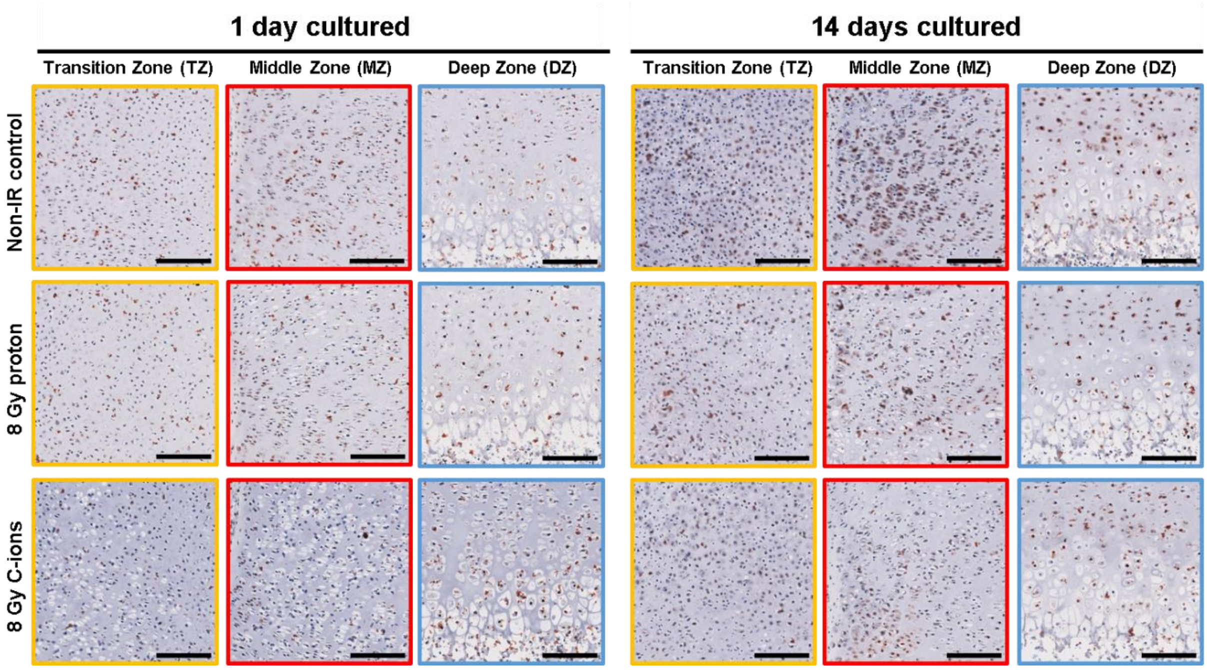

Figure S5. Immunohistochemistry of runt related factor 2 (Runx2). Representative images of Runx2 IHC of *ex vivo* bone cultures exposed to either 8 Gy proton or C-ions radiation with their respective controls followed by subsequent 1- and 14- days *in vitro* (DIV) cultivation. Coloured squares represent transition zone (TZ, yellow), middle zone (MZ, red) and deep zone (DZ, blue). Scale bar - TZ/MZ/DZ = 150 μm.

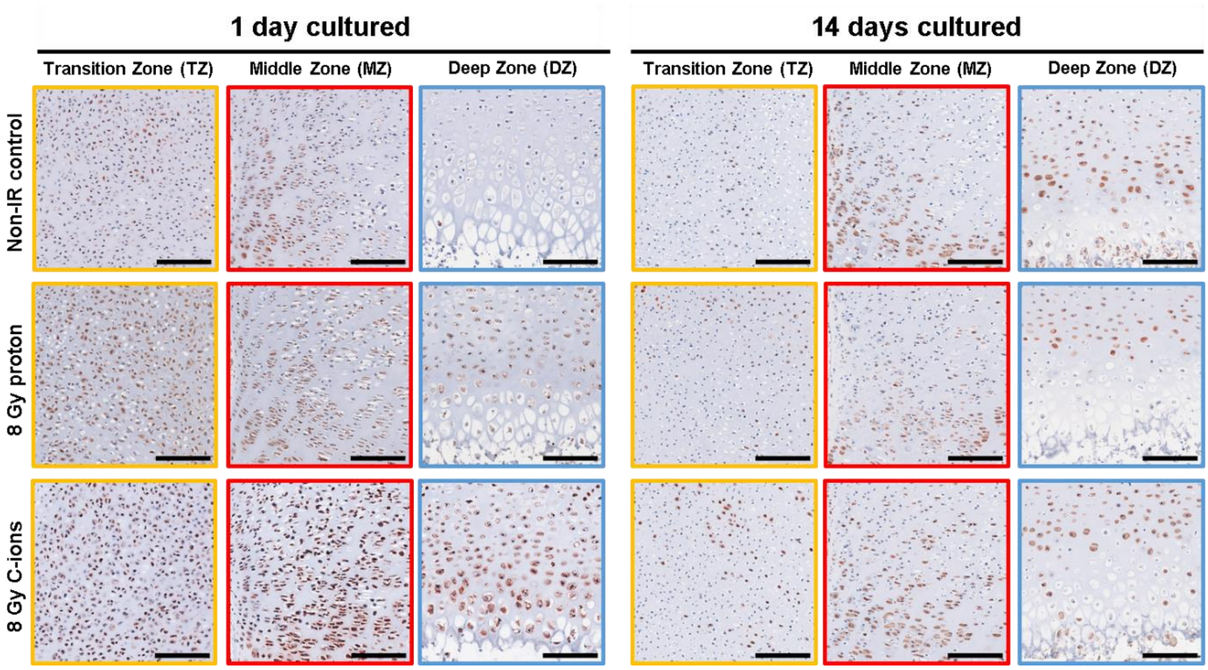

Figure S6. Immunohistochemistry of Osteopotegerin (OPG). Representative images of OPG IHC of *ex vivo* bone cultures exposed to either 8 Gy proton or C-ions radiation with their respective controls followed by subsequent 1- and 14- days *in vitro* (DIV) cultivation. Coloured squares represent transition zone (TZ, yellow), middle zone (MZ, red) and deep zone (DZ, blue). Scale bar - TZ/MZ/DZ = 150 μm.
